# Automated mitosis detection in stained histopathological images using Faster R-CNN and stain techniques

**DOI:** 10.1515/jib-2024-0049

**Published:** 2025-06-11

**Authors:** Jesús García-Salmerón, José Manuel García, Gregorio Bernabé, Pilar González-Férez

**Affiliations:** Faculty of Computer Science, Computer Engineering Department, University of Murcia, Murcia, Spain, https://www.um.es/web/ditec/

**Keywords:** artificial intelligence, cancer, MIDOG challenge, object detection, tumor prognosis

## Abstract

Accurate mitosis detection is essential for cancer diagnosis and treatment. Traditional manual counting by pathologists is time-consuming and may cause errors. This research investigates automated mitosis detection in stained histopathological images using Deep Learning (DL) techniques, particularly object detection models. We propose a two-stage object detection model based on Faster R-CNN to effectively detect mitosis within histopathological images. The stain augmentation and normalization techniques are also applied to address the significant challenge of domain shift in histopathological image analysis. The experiments are conducted using the MIDOG++ dataset, the most recent dataset from the MIDOG challenge. This research builds on our previous work, in which two one-stage frameworks, in particular on RetinaNet using fastai and PyTorch, are proposed. Our results indicate favorable F1-scores across various scenarios and tumor types, demonstrating the effectiveness of the object detection models. In addition, Faster R-CNN with stain techniques provides the most accurate and reliable mitosis detection, while RetinaNet models exhibit faster performance. Our results highlight the importance of handling domain shifts and the number of mitotic figures for robust diagnostic tools.

## 1 Introduction

The incidence of cancer is rising significantly and has become a major global health concern because of its severe and lasting effects on individuals and society. Histopathological analysis, a fundamental element of cancer diagnosis, is essential for detecting tumors and developing effective treatment plans. During the examination of histopathological images, recognizing mitosis is a crucial task for evaluating cancer and forecasting its progression. Currently, skilled pathologists perform this task manually by examining Hematoxylin and Eosin (H&E) stained tissue sections under a microscope. However, this traditional method is time-consuming, prone to errors, and exhibits significant variability between observers [1].

In recent years, various *Machine Learning* (ML) techniques have advanced and gained popularity in the field of histopathology, with one prominent approach being *Deep Learning* (DL). This method has experienced rapid advancements, with the development of techniques that rival or even surpass human experts in certain tasks. The incorporation of these techniques into computer-aided diagnostics brings notable advantages, such as the optimization of tasks with significant observer variability that increases diagnostic reliability and minimizing bias [2]. It also benefits for routine quantitative tasks, allowing for a more efficient diagnostic process [2]. These benefits underscore the growing need for mitosis detection methods automatized with DL.

A vital factor in accelerating diagnoses and ensuring accurate grading is the implementation of automated mitosis detection methods. These techniques facilitate the development of personalized treatment plans and have the potential to reduce cancer mortality rates. By improving accuracy, automated detection reduces the tediousness of manual counting, while also offering additional advantages, such as independent mitotic activity scoring and aiding pathologists in pinpointing areas with the highest levels of mitotic activity [1], 3].

Deep Learning methods for image analysis can encounter a significant challenge, their performance may deteriorate when there is a discrepancy between the visual representation of training images and testing images. This issue, known as domain shift, poses difficulties in histopathology due to variations in staining techniques, imaging devices, and tumor types. Humans can adjust to these variations, whereas Machine Learning models often find it difficult to adapt.

To address the problem of domain shift, the *MItosis DOmain Generalization* (MIDOG) 2021 challenge was established. The MIDOG challenge is designed to tackle the domain shift caused by variations in *Whole Slide Image* (WSI) scanners, which can significantly alter colour representation that is essential for detecting mitotic figures. Through participation in this challenge, researchers seek to improve the adaptability of automated mitosis detection methods across various environments, thereby increasing their effectiveness in diagnostic applications [2]. Due to the popularity and impact of the MIDOG 2021 challenge and the interest in other tumor types, the MIDOG 2022 challenge [4] emerged. MIDOG 2022 is an enhancement of MIDOG 2021 by including new tumor type and 405 training images. Afterwards, the MIDOG 2022 challenge was extended, leading to the creation of MIDOG++, which is the latest development from the MIDOG challenge. This new dataset contains 503 histological images from seven different tumor types with varying morphologies. A detailed explanation of MIDOG++ dataset is provided in Section 3.1 since we use this dataset in our work.

In our previous work [5], we replicate and validate MIDOG++ work with RetinaNet using fastai and also propose a RetinaNet model using PyTorch. Results for this former work prove the effectiveness of one-stage object detection models, such as RetinaNet, in mitosis detection within histopathological images.

Since one-stage object detection models are less accurate but more efficient compared to two-stage models [6], we want to analyze the behavior and performance of a two-stage object detection model in mitosis detection within MIDOG++ dataset. Results could show that a two-stage model is suitable for tasks requiring precise detection when time is not critical. In particular, we choose Faster R-CNN as two-stage model because it demonstrates the best performance within the R-CNN family and represents the latest iteration of this architecture. Faster R-CNN builds upon the strengths of its predecessors, R-CNN and Fast R-CNN, by integrating the *Region Proposal Network* (RPN) directly into the mode [7].

In addition, we want to address *Domain Shift* (DS), since it is considered a challenge for the medical analysis research community, specially for the computational pathology community. Domain shift arises when discrepancies exist in data distribution between the source and target domains. These variations complicates the direct application of trained models to previously unseen data. The problem is that histopathological images usually can come from different sources, such as scanners or hospital, or even different in staining protocols, and images may have significant variability among them. In order to tackle this issue, in this work we apply stain normalization and stain augmentation technique to our proposed model.

Therefore, this current research, that extends our previous work [5], has two main objectives: first, to implement a two-stage object detection model, and second, to apply innovative techniques to tackle the challenge of domain shift in this field.

Results show that our two-stage object detection model based on Faster R-CNN usually achieves superior F1-scores and detection accuracy than our previous one-stage models, although our one-stage models show better *Average Precision* (AP) and faster inference times. In addition, the use of stain techniques improve model generalization across different tumor types but increased training times. Therefore, Faster R-CNN model with stain techniques provides the best accuracy.

## 2 Related work

In this section, we firstly outline the mitosis detection techniques, and then the specifics of the object detection models, and finally the techniques for addressing domain shift.

### 2.1 Mitosis detection techniques

The mitosis detection methodologies can be classified into three categories [1]. The first one, called handcrafted features methods extracts manually features from data and trains Machine Learning algorithms to recognize or classify patterns. Initially, the input image is processed to detect candidate cells or nuclei. Then, there is a feature extraction stage. Finally, there is a extraction and classification stage for the candidate cells. This stage generally uses standard algorithms such as *Support Vector Machines* (SVM), *Random Forest* (RF), *Linear Discriminant Analysis* (LDA) or *Multi Layer Perceptron* (MLP). These algorithms classify the candidate cells into mitotic cell or non-mitotic cell, thus completing the mitosis detection process [1].

The second method is based on Deep Learning. Nowadays, Deep Learning methods have become extensively used in medical systems for image-processing tasks, including mitosis detection, cell nucleus segmentation and tissue classification. Detection methods based on DL exploits the abilities of neural network self-learning to automatically extract features and train features. To tackle the challenges of mitosis detection, methods that utilize *Deep Convolutional Neural Network* (DCNN) are commonly employed due to their effectiveness in achieving accurate results [1], 8]. Approaches based on *Convolutional Neural Network* (CNN) are also particularly notable in this regard. CNN-based methods are widely used in medical image analysis as they improve computer vision tasks, including image classification [9], object detection [7], 10], semantic segmentation [11] and instance segmentation [12]. In our research, we employ Deep Learning models, specifically CNN-based methods, to tackle challenges in mitosis detection and improve medical image analysis, with a focus on object detection.

The third one is a combination of both methods. Methodologies for mitosis detection utilize either layered handcrafted features or features extracted through CNNs. Nevertheless, using only handcrafted features results in low detection accuracy, while CNN-based techniques are limited by their computational complexity [13]. The integration of both methodologies can potentially enhance the overall performance of the mitosis detection system.

### 2.2 Object detection models

Object detection is a task within computer vision that involves identifying objects in an image by determining both their category and location. This process not only classifies the objects but also predicts their position using bounding boxes [14]. As a result, the aim of object detection is twofold: locating objects in the image (object localization) and assigning them to the correct category (object classification).

Recently, there has been growing interest in object detection tasks, particularly within the field of histopathology [15] and mitosis detection [8], 16]. The models are generally divided into two main categories: one-stage and two-stage approaches. In general terms, two-stage models typically achieve superior accuracy but demand higher computational resources compared to one-stage models. The accuracy-computational trade-off is heavily influenced by the choice of the backbone network and the hyperparameter configuration [17].

DCNN are the backbone network for object detection models. To improve feature representation performance continue, network architectures become increasingly complex, with deeper layers and more parameters. Consequently, networks known as *Complex Backbone Network* (CBN) has been proposed. However, in environments with limited computing power and storage, *Lightweight Backbone Network* (LBN) structures are used to simplify the network structure without compromising accuracy [6].

In addition, to enhance accuracy, the depth of Complex Backbone Networks has been increased. Some examples are VGGNet [18], GoogLeNet [19] and ResNet [9]. All of this DCNN came up after the success of AlexNet [20], known as the first CNN.

One-stage models perform region proposal and classification in a single stage. A single feed-forward fully CNN directly outputs both the bounding boxes and the object classification. RetinaNet and *You Only Look Once* (YOLO) [21] are examples of these models.

Two-stage frameworks split the detection process into two stages: region proposal and classification. Firstly, these models utilize reference boxes known as anchors and generate multiple object candidates, referred to as *Region of Interest* (RoI). Subsequently, the proposed regions are classified, and their localization is refined. Examples of the two-stage approaches most commonly used are R-CNN (Region-based Convolutional Neural Network), Fast R-CNN, Faster R-CNN and Cascade R-CNN [14].

Since we are using Faster R-CNN, we explain this model in more detail. Faster R-CNN consists of a single and unified network for object detection, its consists of two modules. The first module is a DCNN that proposes regions and the second module is the Fast R-CNN detector [22] that uses the proposed regions. Faster R-CNN utilizes the recently popular terminology of neural networks with “attention” [23] mechanisms, the Region Proposal Network module tells the Fast R-CNN module where to look.

Furthermore, other studies have demonstrated the efficiency of Faster R-CNN for mitosis detection in histopathological images [16], 24], 25]. Focusing on how this model performs detection on various datasets different from the one we will use, and how stain techniques enhance the model’s performance. However, our study employs a novel and more complex dataset, along with a different staining technique implementation, to address the domain shift problem in the dataset.

### 2.3 Techniques for addressing domain shift

DS is considered a challenge for the computational pathology community. Domain shift occurs when there are discrepancies in data distribution between the source and target domains, making the direct applicability of trained models to unseen data difficult. Deep Learning models in computational pathology have demonstrated vulnerability to domain shift, as well as typical corruptions and perturbations [26], 27].

Among all the domain generalization methods developed, in this work we apply stain normalization and stain augmentation. Stain normalization serves as a preprocessing stage, this method aims to correct inconsistencies in the colours of histological images resulting from different staining procedures and differences between scanners [28]. Stain augmentation methods aim to generate new images to enhance robustness to colour variations, under the assumption that objects of interests are invariant to changes in colour intensity and illumination [29]. Various studies [29], [30], [31] have shown that stain techniques effectively address the domain shift problem, and that applying stain augmentation after stain normalization achieves better results than using stain normalization alone.

## 3 Design and implementation

In this research, we have two main objectives. The first one is to develop a two-stage object detection model, specifically Faster R-CNN. The second is to explore and implement novel image processing techniques aimed at addressing the issue of domain shift, a common challenge in medical imaging. The code we developed is available at: https://github.com/jesussgs/faster-midog-plus.

### 3.1 Dataset

**Figure 1:**
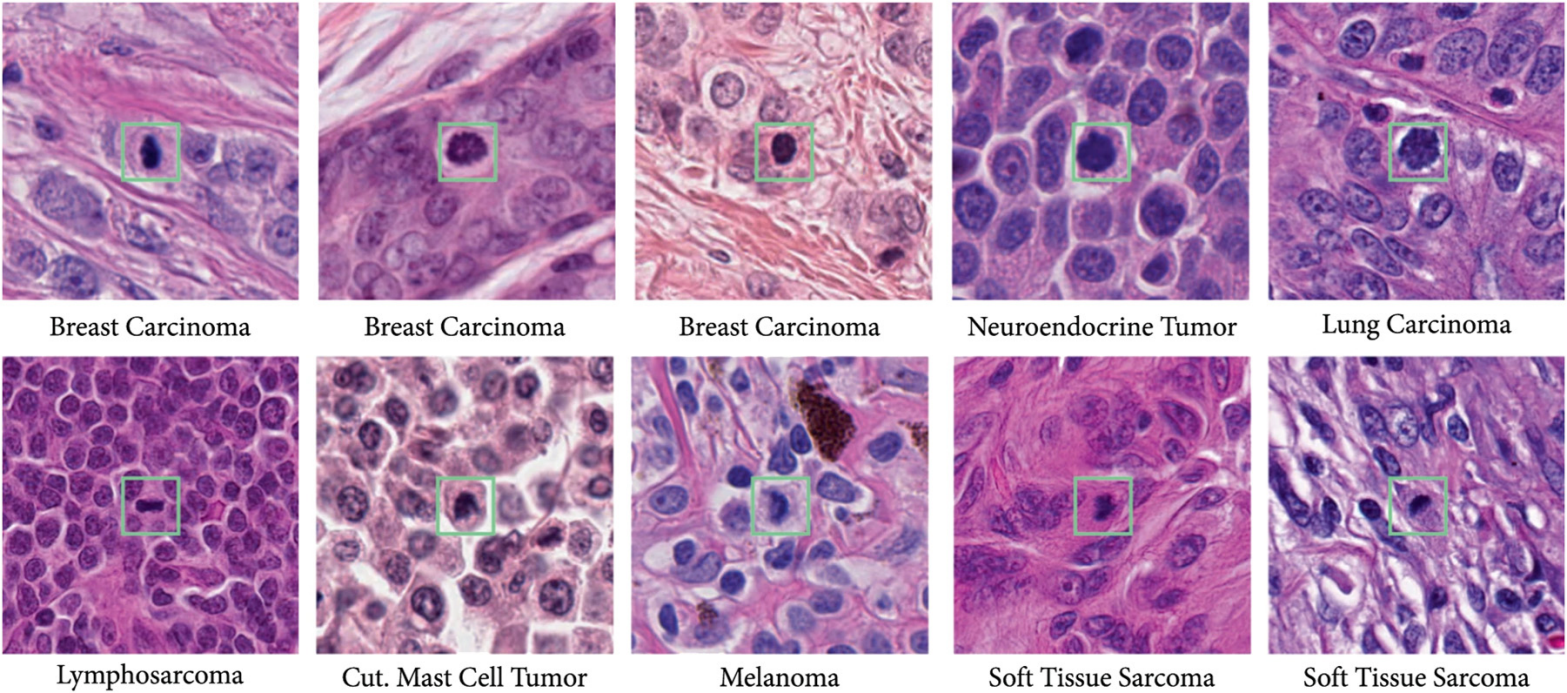
Mitotic figures candidates from all domains [32]. Note that *h* stands for human, *c* for canine, *carci* for carcinoma, *t* for tumor, *cut* for cutaneous and *sarc* for sarcoma.

In our experiments, we utilize the MIDOG++ dataset [32], the most recent dataset from the original MIDOG challenge [2]. MIDOG++ expands the MIDOG dataset with the addition of new images and/or annotations for more cases, and two additional tumor types: canine soft tissue sarcoma and human melanoma. Figure 1 presents examples of mitotic figures from this extended dataset.

MIDOG++ stands out by providing images across a diverse array of domains, with a strong focus on various tumor types. In fact, this is the first dataset that encompasses multiple sources of domain shifts that are crucial for pathological diagnosis [32].

The MIDOG++ dataset includes region-of-interest images from 503 histological specimens representing seven distinct tumor types with diverse morphologies: breast carcinoma, lung carcinoma, lymphosarcoma, neuroendocrine tumor, cutaneous mast cell tumor, cutaneous melanoma, and (sub)cutaneous soft tissue sarcoma. Both human and canine samples were processed and stained at various human and veterinary pathology laboratories using standard Hematoxylin and Eosin (H&E) staining. The images were digitized by one of five whole slide scanners, each at either 0.23 μm/px or 0.25 μm/px resolution. The dataset contains labels for 11,937 mitotic figures, which were distinguished from 14,351 imposter cells. The labeling process involved a blinded consensus by two pathologists, with a final review by a third pathologist for any disagreements [32].

### 3.2 Implementation of Faster R-CNN

In order to compare our previous results developed with one-stage models [5], here we implement a two-stage object detection model, namely Faster Region-based Convolutional Neural Network (Faster R-CNN). We choose Faster R-CNN since it is a widely-used in other studies related to mitosis detection [16], 33], 34]. Two-stage models generally achieve higher confidence predictions and better results compared to one-stage models, but they come with significantly higher computational costs and time-complexity during training and inference. For the implementation of Faster R-CNN we use PyTorch due to its widespread use and scalability.

The experiments are conducted across two distinct domains: single-domain and leave-one-out. The single-domain approach trains the model on a single tumor type and evaluates it across all tumor types. In the leave-one-out approach, the model is trained on all tumor types except one and then it is evaluated on all tumor types.

We adapt the training and evaluation procedure to use PyTorch for the utilization of Faster R-CNN model. This training process involves adapting the code from the fastai framework to PyTorch, integrating additional libraries such as Albumentations to ensure precise data transformations, and adding custom classes and elements to facilitate an effective training stage. Our proposed Faster R-CNN model also undergoes fine-tuning and utilizes a ResNet-50-FPN backbone. In particular, our model represents an improved version of the conventional pretrained Faster R-CNN model. Note that using a denser backbone offers benefits like improved precision and generalization but comes with drawbacks such as longer training times and higher computational costs.

For the evaluation stage, we adjust the process to rely solely on the PyTorch framework and revised the implementation of *Non-Maximum Suppression* (NMS). The results are primarily evaluated using the F1-score, the key metric in the MIDOG challenge.

(1) $$\text{F}1=\frac{2\times \text{precision}\times \text{recall}}{\text{precision}+\text{recall}}=\frac{2\times TP}{2\times TP+FP+FN}$$

The F1-score has range [0,1] and is calculated using Equation (1), where *TP* represents true positives, *FP* are false positives and *FN* are false negatives.

### 3.3 Applying stain augmentation and normalization techniques

In order to tackle the domain shift in the MIDOG++ [32] dataset, we apply stain normalization and stain augmentation technique to our proposed model. We select them due to two main factors. First, stain normalization helps to standardize the variability in visual features, allowing the models to become less sensitive to these inconsistencies and focus on more critical image characteristics. Second, stain augmentation expands the dataset by introducing variations in colour intensity and staining patterns, enhancing the models’ robustness to unseen variations in new domains. We rely on existing implementations of these stain techniques [30], 35] and are not introducing any new methods.

Stain normalization technique addresses stain variation in digital pathology, including the MIDOG challenge [2]. The Vahadane stain normalization method is commonly used since it preserves the structural properties of stained tissue samples and it is robust to stain sparsity in pathology images. Additionally, Vahadane stain normalization has been shown to be superior to other state-of-the-art methods [28].

Vahadane stain normalization employs *Sparse Non-negative Matrix Factorization* (SNMF) to estimate the stain matrix (*S*) and concentration matrix (*C*) from both source and target images. It then scales the concentration map of the source image and combines it with the stain matrix of the target image to achieve normalization [30].

Our model is enhanced by incorporating stain augmentation in training, that involves randomly altering the concentration of H&E stains in the source image. Using the SNMF algorithm, we first extract the source stain matrix (*S*) and concentration matrix (*C*). We then scale and shift the stain concentrations, and finally convert the modified stain information back to RGB space, resulting in an augmented image, denoted as *Î* [36].

(2) $$\hat{I}=I_{0}\exp\left(-S\left(\alpha C+\beta \right)\right)$$

The augmented image *Î* is created according to Equation (2), where *I*_0_ represents the incident intensity of the light source derived from the source image *I*. Here, *α* ∼ *U*(0.75, 1.25) and *β* ∼ *U*(−0.2, 0.2) are the stain concentration scale and shift factors, respectively, which are randomly selected from uniform distributions [36].

We perform stain normalization and stain augmentation simultaneously by setting the *S* matrix in Equation (2) to a pre-extracted target stain matrix. The target stain matrix is obtained by setting a target image and extracting its stain matrix. In our experiments, we utilize *“009.tiff”* as the target image due to its comprehensive H&E stain colour spectrum. Furthermore, previous works related to the MIDOG challenge have demonstrated its effectiveness for stain augmentation techniques [36].

In our implementation, we utilize TIAToolbox [35] library to realize both stain normalization and stain augmentation techniques.

We first extract the stain matrix from the target image. Then by using TIAToolbox, we integrate the stain normalization and stain augmentation techniques into the Albumentations pipeline developed. Finally, we repeat the experiments with our proposed models to evaluate their performance. We use this new pipeline to train Faster R-CNN with PyTorch, while maintaining the same training configuration of both models.

## 4 Results

All implementations are developed using Python 3.8, and PyTorch 1.13.0 with torchvision 0.14.0. Experiments are conducted on a system powered by a dual AMD EPYC 7282 CPU and a 128 GiB DDR-4 DRAM. This system is equipped a Nvidia GeForce RTX 4090 GPU with 24 GB GDDR6X memory. All experiments leverage the computational capabilities of the RTX 4090, utilizing the GPU for processing.

### 4.1 Analysis of Faster R-CNN

For Faster R-CNN, in the training phase, the average epoch duration is 63.72 s. Additionally, the average inference time per image is 5.65 s for Faster R-CNN.

For both average training time and average inference time, we observe higher times for Faster R-CNN compared to our previous research [5]. This time increase is due to Faster R-CNN architecture. As a two-stage approach, Faster R-CNN first performs an object proposal stage, and then a classification and regression stage, these two stages increase its complexity. In addition, we are using a denser backbone (ResNet-50-FPN) that implies longer training times and higher computational costs.

**Figure 2:**
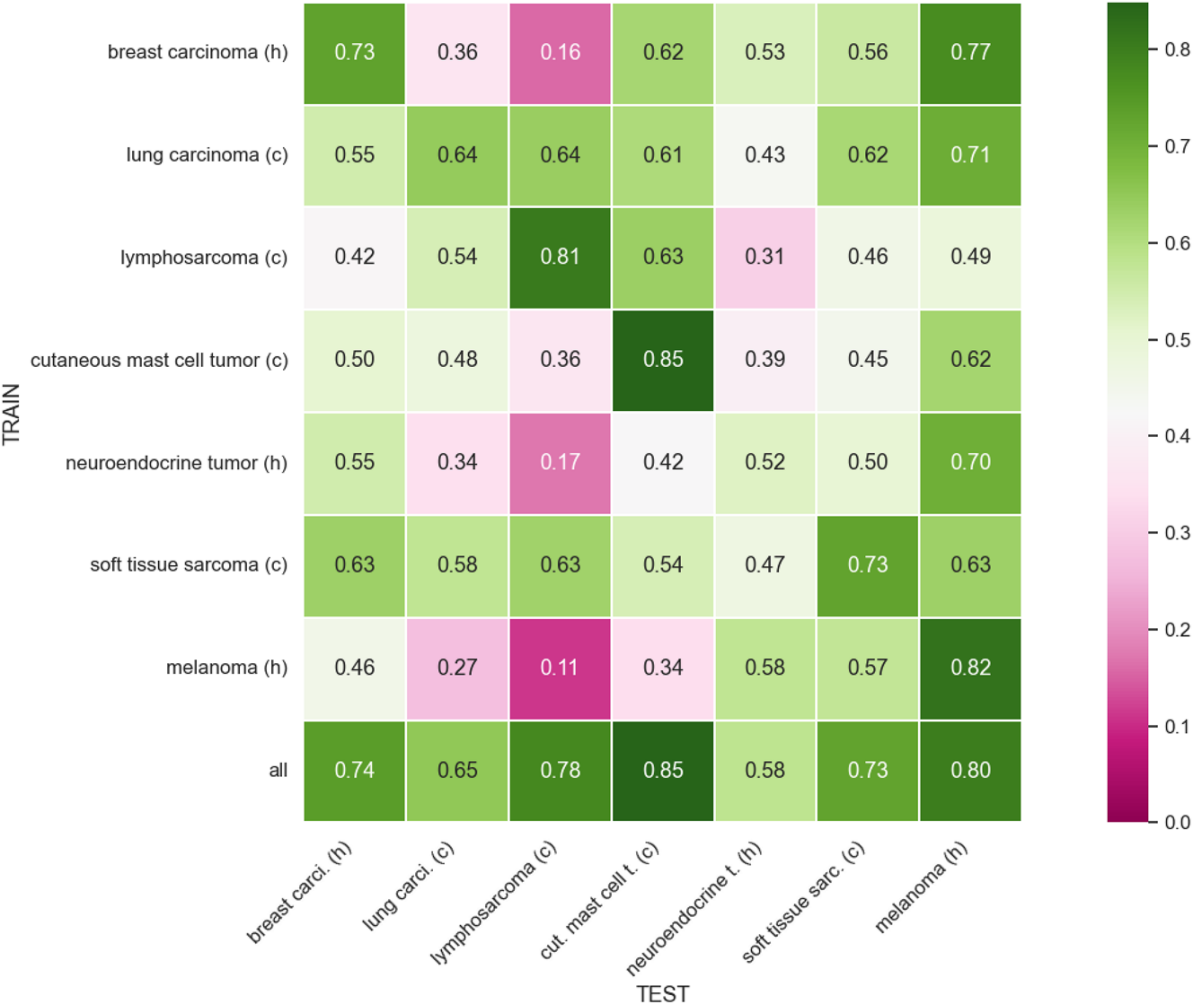
Domain matrix for single-domain training for Faster R-CNN. Note that *h* stands for human, *c* for canine, *carci* for carcinoma, *t* for tumor, *cut* for cutaneous and *sarc* for sarcoma.

We now analyze the results of Faster R-CNN in terms of the F1-score. Figure 2 shows the mean F1-score for each tumor type in each single-domain experiment using Faster R-CNN. The diagonal generally presents higher F1-scores compared to off-diagonal experiments. This is expected, as it shows the model performs best when trained and tested on the same cancer type. For example, human breast carcinoma achieves a strong F1-score when trained and tested on the same type (diagonal), and it also demonstrates good generalization when tested on human melanoma. In addition, canine cutaneous mast cell tumor achieves the highest F1-score of 0.85. However, human neuroendocrine tumor struggles to obtain high F1-scores, and canine lung carcinoma and canine soft tissue sarcoma only achieve good scores when trained with all tumor types. These low scores can be attributed to two main factors: the quantity of mitotic figures associated with each specific tumor type, and the morphological similarities between different tumor types, that limit the model’s generalization capabilities when trained on a single tumor type. Nevertheless, in all tumor types experiment, the primary reason for the low F1-scores remains the limited number of mitotic figures. Table 1 shows the mean and standard deviation of the F1-score achieved using Faster R-CNN in each single-domain experiment.

**Table 1:** Mean and standard deviation of F1-score for single-domain training for Faster R-CNN.

	Breast carcinoma	Lung carcinoma	Lymphosarcoma	C. mast cell tumor	Neuroendocrine tumor	Soft tissue sarcoma	Melanoma
Breast carcinoma	0.73 ± 0.01	0.36 ± 0.15	0.16 ± 0.08	0.62 ± 0.08	0.53 ± 0.06	0.56 ± 0.05	0.77 ± 0.03
Lung carcinoma	0.55 ± 0.03	0.64 ± 0.01	0.64 ± 0.04	0.61 ± 0.04	0.43 ± 0.06	0.62 ± 0.03	0.71 ± 0.04
Lymphosarcoma	0.42 ± 0.05	0.54 ± 0.02	0.81 ± 0.01	0.63 ± 0.02	0.31 ± 0.07	0.46 ± 0.06	0.49 ± 0.08
C. mast cell tumor	0.50 ± 0.06	0.48 ± 0.04	0.36 ± 0.06	0.85 ± 0.01	0.39 ± 0.10	0.45 ± 0.10	0.62 ± 0.07
Neuroendocrine tumor	0.55 ± 0.11	0.34 ± 0.14	0.17 ± 0.07	0.42 ± 0.08	0.52 ± 0.03	0.50 ± 0.09	0.70 ± 0.04
Soft tissue sarcoma	0.63 ± 0.04	0.58 ± 0.04	0.63 ± 0.06	0.54 ± 0.08	0.47 ± 0.01	0.73 ± 0.01	0.63 ± 0.04
Melanoma	0.46 ± 0.08	0.27 ± 0.07	0.11 ± 0.05	0.34 ± 0.14	0.58 ± 0.03	0.57 ± 0.08	0.82 ± 0.01
All	0.74 ± 0.01	0.65 ± 0.03	0.78 ± 0.01	0.85 ± 0.01	0.58 ± 0.03	0.73 ± 0.02	0.80 ± 0.03

**Figure 3:**
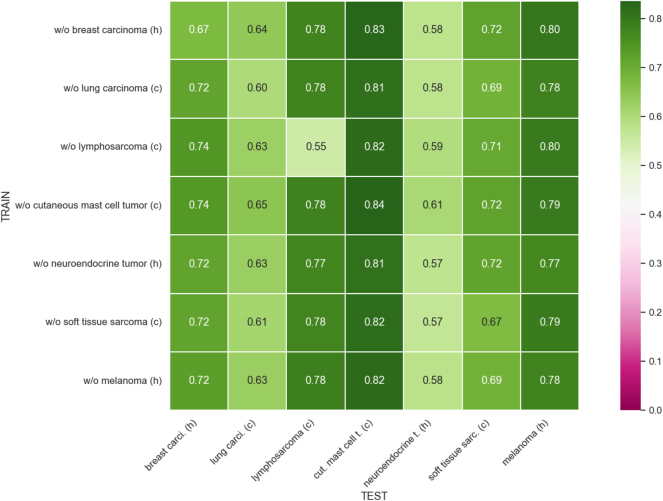
Domain matrix for leave-one-out training for Faster R-CNN. Note that *h* stands for human, *c* for canine, *carci* for carcinoma, *t* for tumor, *cut* for cutaneous and *sarc* for sarcoma.

Figure 3 shows the mean F1-score for each tumor type in each leave-one-out experiment using Faster R-CNN. We observe significant generalization across all experiments when leaving one tumor type out for training. Notably, the canine cutaneous mast cell tumor achieves the highest F1-score of 0.84, with canine lymphosarcoma and human melanoma also yielding strong results. However, the human neuroendocrine tumor type shows the lowest F1-scores, likely due to the smaller number of mitotic figures and images compared to the other tumor types. Table 2 shows the mean and standard deviation of the F1-score achieved using Faster R-CNN in each leave-one-out experiment.

**Table 2:** Mean and standard deviation of F1-score for leave-one-out training for Faster R-CNN.

	Breast carcinoma	Lung carcinoma	Lymphosarcoma	C. mast cell tumor	Neuroendocrine tumor	Soft tissue sarcoma	Melanoma
w/o Breast carcinoma	0.67 ± 0.06	0.64 ± 0.02	0.78 ± 0.03	0.83 ± 0.03	0.58 ± 0.01	0.72 ± 0.02	0.80 ± 0.02
w/o Lung carcinoma	0.72 ± 0.01	0.60 ± 0.01	0.78 ± 0.01	0.81 ± 0.01	0.58 ± 0.03	0.69 ± 0.03	0.78 ± 0.00
w/o Lymphosarcoma	0.74 ± 0.01	0.63 ± 0.01	0.55 ± 0.11	0.82 ± 0.02	0.59 ± 0.04	0.71 ± 0.02	0.80 ± 0.01
w/o C. mast cell tumor	0.74 ± 0.02	0.65 ± 0.01	0.78 ± 0.01	0.84 ± 0.01	0.61 ± 0.03	0.72 ± 0.01	0.79 ± 0.01
w/o Neuroendocrine tumor	0.72 ± 0.01	0.63 ± 0.01	0.77 ± 0.01	0.81 ± 0.02	0.57 ± 0.03	0.72 ± 0.02	0.77 ± 0.02
w/o Soft tissue sarcoma	0.72 ± 0.04	0.61 ± 0.04	0.78 ± 0.00	0.82 ± 0.01	0.57 ± 0.03	0.67 ± 0.03	0.79 ± 0.01
w/o Melanoma	0.72 ± 0.03	0.63 ± 0.01	0.78 ± 0.01	0.82 ± 0.02	0.58 ± 0.04	0.69 ± 0.04	0.78 ± 0.02

As Section 4.3 shows, results obtained by Faster R-CNN surpass those obtained in our previous research [5]. This demonstrates that a two-stage approach model, despite their higher cost in time and computation, achieves better results than a one-stage model in MIDOG++ dataset.

### 4.2 Analysis of stain augmentation and normalization techniques

We now evaluate and analyze results after implementing the stain augmentation and stain normalization for Faster R-CNN. During the training phase, there is an increase in the average epoch duration due to the addition of the stain techniques. However, the average inference time remains unaffected by this additional processing.

**Figure 4:**
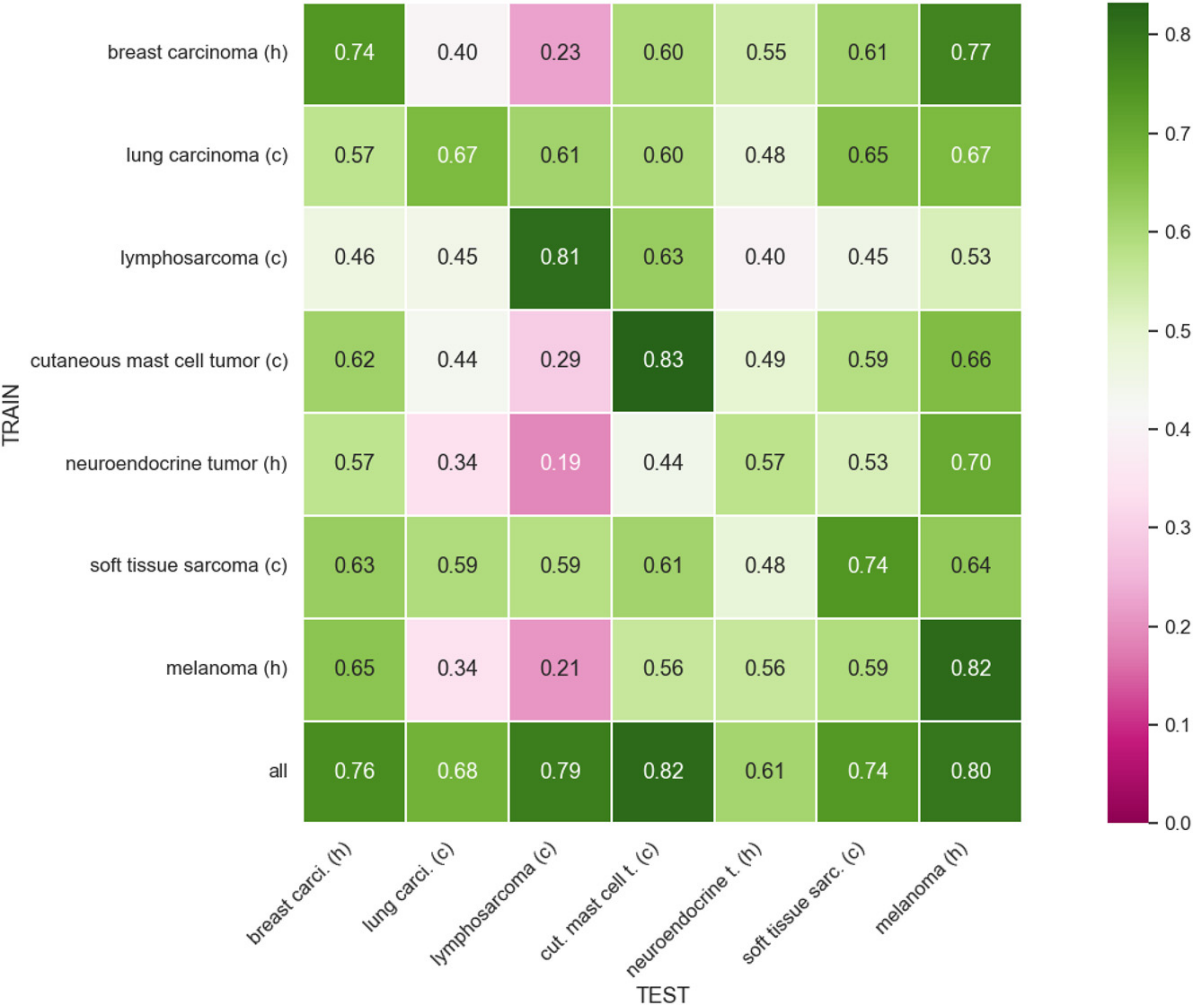
Domain matrix for single-domain training for Faster R-CNN with stain techniques. Note that *h* stands for human, *c* for canine, *carci* for carcinoma, *t* for tumor, *cut* for cutaneous and *sarc* for sarcoma.

Figure 4 presents the mean F1-score for each tumor type in each single-domain experiment using Faster R-CNN with stain techniques. We observe that results have improved in most cases, highlighting the achievement of better generalization in all experiments. Regarding the diagonal results, F1-scores have improved in all cases, except for canine cutaneous mast cell tumor, which has slightly decreased from 0.85 to 0.83 while still maintaining the highest F1-score, and human melanoma, which has remained the same F1-score. When training with all tumor types, we appreciate a similar pattern as with the diagonal results: each tumor type has slightly improved except for canine cutaneous mast cell tumor, which has decreased from 0.85 to 0.82, and human melanoma, which has remained unchanged. Table 3 shows the mean and standard deviation of the F1-score achieved using Faster R-CNN in each sigle-domain experiment with stain techniques.

**Table 3:** Mean and standard deviation of F1-score for single-domain training for Faster R-CNN with stain techniques.

	Breast carcinoma	Lung carcinoma	Lymphosarcoma	C. mast cell tumor	Neuroendocrine tumor	Soft tissue sarcoma	Melanoma
Breast carcinoma	0.74 ± 0.01	0.40 ± 0.05	0.23 ± 0.05	0.60 ± 0.08	0.55 ± 0.02	0.61 ± 0.02	0.77 ± 0.01
Lung carcinoma	0.57 ± 0.09	0.67 ± 0.01	0.61 ± 0.03	0.60 ± 0.08	0.48 ± 0.05	0.65 ± 0.03	0.67 ± 0.06
Lymphosarcoma	0.46 ± 0.09	0.45 ± 0.03	0.81 ± 0.00	0.63 ± 0.05	0.40 ± 0.13	0.45 ± 0.07	0.53 ± 0.10
C. mast cell tumor	0.62 ± 0.04	0.44 ± 0.04	0.29 ± 0.07	0.83 ± 0.01	0.49 ± 0.04	0.59 ± 0.04	0.66 ± 0.04
Neuroendocrine tumor	0.57 ± 0.02	0.34 ± 0.07	0.19 ± 0.04	0.44 ± 0.11	0.57 ± 0.01	0.53 ± 0.03	0.70 ± 0.03
Soft tissue sarcoma	0.63 ± 0.02	0.59 ± 0.03	0.59 ± 0.04	0.61 ± 0.05	0.48 ± 0.04	0.74 ± 0.01	0.64 ± 0.04
Melanoma	0.65 ± 0.03	0.34 ± 0.07	0.21 ± 0.06	0.56 ± 0.06	0.56 ± 0.05	0.59 ± 0.04	0.82 ± 0.02
All	0.76 ± 0.01	0.68 ± 0.01	0.79 ± 0.01	0.82 ± 0.01	0.61 ± 0.01	0.74 ± 0.01	0.80 ± 0.01

**Figure 5:**
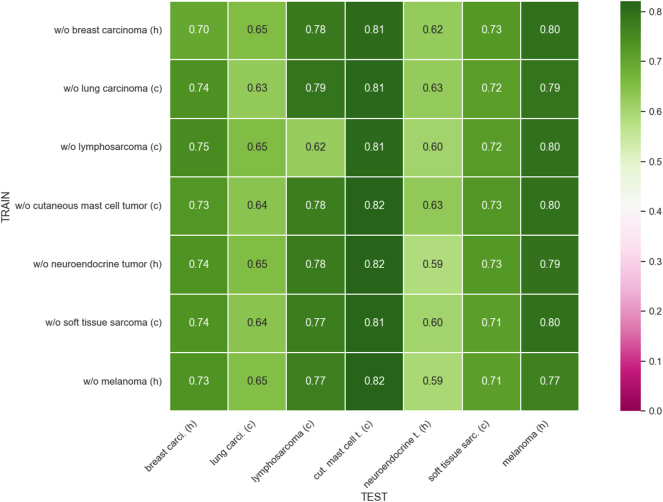
Domain matrix for leave-one-out training for Faster R-CNN with stain techniques. Note that *h* stands for human, *c* for canine, *carci* for carcinoma, *t* for tumor, *cut* for cutaneous and *sarc* for sarcoma.

Figure 5 presents the mean F1-score for each tumor type in each leave-one-out experiment using Faster R-CNN with stain techniques. The improvement in F1-scores across most cases is appreciable. Notably, the generalization of models has enhanced even when predicting tumor types not included in the training set. Among the different tumor types, the F1-scores for human breast cancer, human neuroendocrine tumor, and canine soft tissue sarcoma stand out. Additionally, in the experiment where canine lymphosarcoma is excluded from training, the F1-score for this tumor type improved from 0.55 to 0.62. However, we also observe a slight decrease in the F1-score for canine cutaneous mast cell tumor, with the previous maximum of 0.84 dropping to 0.82. Table 4 shows the mean and standard deviation of the F1-score achieved using Faster R-CNN in each leave-one-out experiment with stain techniques.

**Table 4:** Mean and standard deviation of F1-score for leave-one-out training for Faster R-CNN with stain techniques.

	Breast carcinoma	Lung carcinoma	Lymphosarcoma	C. mast cell tumor	Neuroendocrine tumor	Soft tissue sarcoma	Melanoma
w/o Breast carcinoma	0.70 ± 0.01	0.65 ± 0.02	0.78 ± 0.01	0.81 ± 0.02	0.62 ± 0.03	0.73 ± 0.01	0.80 ± 0.01
w/o Lung carcinoma	0.74 ± 0.02	0.63 ± 0.00	0.79 ± 0.01	0.81 ± 0.01	0.63 ± 0.01	0.72 ± 0.01	0.79 ± 0.01
w/o Lymphosarcoma	0.75 ± 0.01	0.65 ± 0.03	0.62 ± 0.02	0.81 ± 0.02	0.60 ± 0.03	0.72 ± 0.02	0.80 ± 0.01
w/o C. mast cell tumor	0.73 ± 0.01	0.64 ± 0.02	0.78 ± 0.01	0.82 ± 0.01	0.63 ± 0.02	0.73 ± 0.02	0.80 ± 0.02
w/o Neuroendocrine tumor	0.74 ± 0.01	0.65 ± 0.01	0.78 ± 0.01	0.82 ± 0.01	0.59 ± 0.02	0.73 ± 0.02	0.79 ± 0.01
w/o Soft tissue sarcoma	0.74 ± 0.01	0.64 ± 0.02	0.77 ± 0.01	0.81 ± 0.02	0.60 ± 0.01	0.71 ± 0.01	0.80 ± 0.01
w/o Melanoma	0.73 ± 0.02	0.65 ± 0.01	0.77 ± 0.01	0.82 ± 0.01	0.59 ± 0.02	0.71 ± 0.01	0.77 ± 0.01

### 4.3 Overall analysis

Finally, we analyze results obtained from all the experiments conducted in our research, to provide a comprehensive overview of which model performs best. Therefore, we compare results from the models developed in our previous research [5] to those obtained by the models proposed in this study.

We first compare Faster R-CNN to RetinaNet-PyTorch and RetinaNet-fastai [5]. We focus on determining which model performs better in terms of F1-score or AP across different scenarios and cases, without considering stain techniques.

Tables 5 and 6 summarize results for F1-score and AP, respectively, with Faster R-CNN, RetinaNet-PyTorch and RetinaNet-fastai, indicating the number of cases where each model obtains the highest F1-score for single-domain cases, along with the mean, maximum and minimum.

**Table 5:** Summary of mean F1-scores for the first group of models [5].

Model	Single domain				Leave-one-out domain			
	Cases won	Mean F1	Max F1	Min F1	Cases won	Mean F1	Max F1	Min F1
RetinaNet-fastai	15	0.5303	0.8460	0.1310	12	0.7028	0.8288	0.5370
RetinaNet-PyTorch	11	0.4987	0.7996	0.0426	18	0.6866	0.7745	0.4975
Faster R-CNN	30	0.5522	0.8486	0.1120	19	0.7115	0.8357	0.5481

**Table 6:** Summary of mean average precision APs for the first group of models [5].

Model	Single domain				Leave-one-out domain			
	Cases won	Mean AP	Max AP	Min AP	Cases won	Mean AP	Max AP	Min AP
RetinaNet-fastai	31	0.3472	0.6518	0.1310	42	0.4590	0.6608	0.2297
RetinaNet-PyTorch	8	0.2355	0.5508	0.0426	1	0.3488	0.5712	0.1554
Faster R-CNN	17	0.3069	0.5937	0.1120	6	0.3901	0.5839	0.1279

Table 5 shows that Faster R-CNN stands out as the best model by winning the most cases compared to other models across both domains. It achieves the highest mean and maximum F1-scores, and its minimum F1-score is acceptable. Regarding RetinaNet-PyTorch, we note its consistency across both domains in comparison to other models. This is evident in the number of cases where it achieves the highest F1-score in leave-one-out, only one case behind from Faster R-CNN.

Table 6 shows that RetinaNet-fastai achieves the best results and wins most cases, surpassing Faster R-CNN. However, the performance of Faster R-CNN is close to that of RetinaNet-fastai. This difference is primarily due to the detection threshold set for each model. A higher detection threshold tends to make more conservative detections, causing the rejection of predictions that might actually be true positives. Consequently, this can increase false negatives and cause a decrease in recall, resulting in a lower AP score. In the experiments, RetinaNet-fastai’s detection thresholds never exceed 0.65, while Faster R-CNN’s thresholds are typically set higher than 0.9 in all cases. During the evaluation stage, we establish the optimal detection threshold by testing different thresholds and selecting the one that achieves the highest F1-score. We observe that this optimal detection threshold depends on the model used, with two-stage models achieving higher detection thresholds compared to one-stage models. RetinaNet-PyTorch falls slightly behind the other models in terms of AP.

Regarding our implementation with the stain techniques Tables 7 and 8 shows results for F1-score and AP, respectively, with Faster R-CNN, Faster R-CNN (S), RetinaNet-PyTorch and RetinaNet-fastai, indicating the number of cases where each model obtains the highest F1-score for single-domain cases, along with the mean, maximum and minimum. Note that (*S*) stands for models implemented with stain augmentation and normalization.

**Table 7:** Summary of F1-scores for all models [5]. Note that *S* means with stain techniques.

Model	Single domain				Leave-one-out domain			
	Cases won	Mean F1	Max F1	Min F1	Cases won	Mean F1	Max F1	Min F1
RetinaNet-fastai	7	0.5303	0.8460	0.1310	10	0.7028	0.8288	0.5370
RetinaNet-PyTorch	8	0.4987	0.7996	0.0426	10	0.6866	0.7745	0.4975
Faster R-CNN	12	0.5522	0.8486	0.1120	10	0.7115	0.8357	0.5481
Faster R-CNN (S)	26	0.5771	0.8327	0.1891	12	0.7242	0.8210	0.5867

**Table 8:** Summary of average precision (AP) for all models [5]. Note that *S* means with stain techniques.

Model	Single domain				Leave-one-out domain			
	Cases won	Mean AP	Max AP	Min AP	Cases won	Mean AP	Max AP	Min AP
RetinaNet-fastai	27	0.3472	0.6518	0.1310	29	0.4590	0.6608	0.2297
RetinaNet-PyTorch	4	0.2355	0.5508	0.0426	1	0.3488	0.5712	0.1554
Faster R-CNN	7	0.3069	0.5937	0.1120	4	0.3901	0.5839	0.1279
Faster R-CNN (S)	13	0.3260	0.5963	0.1120	10	0.4180	0.5884	0.1188

For F1-score, Table 7 shows that Faster R-CNN with stain techniques achieves the best results among all models, with the highest number of cases and closely obtains the highest mean and maximum F1-scores. Notably, Faster R-CNN also achieves the highest minimum mean F1-score. Nevertheless, the utilization of stain augmentation and normalization techniques improves results for RetinaNet-PyTorch in both domains, but Faster R-CNN without stain techniques still achieves superior results.

For AP, Table 8 shows that, again, RetinaNet-fastai is the best option obtaining always the highest values. The choice of detection thresholds impacts all models in this scenario, since it affects the mean AP score achieved by the models. However, note that Faster R-CNN with stain techniques achieves AP scores close to those of RetinaNet-fastai in single-domain, although it slightly lags behind in leave-one-out experiments.

Finally, we compare the models performance in terms of both training and inference time. Additionally, we analyze the mean detection threshold range of the models, obtained by studying all experiments and the various detection thresholds employed. The summary of all models performance are shown in Table 9.

**Table 9:** Summary of all models performance [5].

Model	Average training time	Average inference time	Mean detection threshold range
RetinaNet-fastai	42.66	1.34	[0.5, 0.61]
RetinaNet-PyTorch	43.33	2.43	[0.53, 0.65]
Faster R-CNN	63.72	5.65	[0.88, 0.96]
Faster R-CNN (S)	122.70	5.71	[0.9, 0.98]

In terms of average training time per epoch, RetinaNet models achieve lower training times compared to Faster R-CNN. This is attributed to their one-stage architecture, which contrasts with the two-stage architecture of Faster R-CNN. Furthermore, the application of the stain techniques increases the average training time of models. In terms of average inference time per image, one-stage models performs faster inferences compared to two-stage models, and RetinaNet-fastai presents the lowest average inference time. This can be attributed to its lighter ResNet backbone [18] compared to the one used by RetinaNet-Pytorch [5]. The application of stain techniques does not affect in average inference time of models.

Regarding the mean detection threshold range, we observe a notable contrast between the thresholds used by one-stage models and those used by two-stage models. One-stage models commonly set detections threshold that never exceeds 0.7. Among these one-stage models, we appreciate how RetinaNet-PyTorch tends to use higher detection thresholds than RetinaNet-fastai. On the other hand, we observe how two-stage models employs high detection thresholds, often approaching or exceeding 0.9. Furthermore, we notice how the utilization of stain augmentation and normalization tends to make more conservative detections using a higher threshold that models without stain techniques. Notably, the Faster R-CNN model with stain techniques achieves the highest range of detection threshold reaching even 0.98 in different cases.

As a summary, Faster R-CNN with stain augmentation and normalization is the top choice due to its higher detection thresholds, despite longer training and inference times. It provides more reliable predictions compared to RetinaNet. However, if speed is prioritized, RetinaNet is preferable.

In terms of results, Faster R-CNN with stain techniques generally provides the highest F1-scores, which is crucial since F1-score is the main metric in MIDOG challenges. While RetinaNet-fastai achieves the best AP, Faster R-CNN is close behind, particularly in single-domain scenarios. However, its conservative detection approach can slightly lower AP by increasing false negatives. During inference, we observe that Faster R-CNN models generated more predictions compared to the other models, particularly the variant with stain augmentation and normalization, further supporting the application of these Faster R-CNN models. Finally, model performance is impacted by the variability in mitotic figures and the amount of images from different tumor types. Notably, canine lung cancer and human neuroendocrine tumors achieve the lowest results, due to the lower number of mitotic figures present in their images.

## 5 Conclusion and future work

In this research, we utilize the MIDOG++ [32] dataset to implement and evaluate the performance of two-stage object detection models, specifically Faster R-CNN, with and without the application of stain augmentation and normalization techniques. The primary objective is to demonstrate the effectiveness of two-stage object detection models in automated mitosis detection. The second one is to evaluate the role of stain augmentation and normalization in mitigating domain shift.

Faster R-CNN models, especially with stain techniques, achieve superior F1-scores and detection accuracy, while RetinaNet shows better AP and faster inference times. The use of stain techniques improves model generalization across different tumor types but increased training times. However, models struggle to detect mitosis in tumors with fewer mitotic figures, such as neuroendocrine tumor and canine lung cancer.

As future work, we plan to further refine stain techniques to more effectively address covariate shift, which remains a critical challenge in improving model generalization across different histopathological domains. Additionally, we aim to explore alternative models like *DEtection TRansformer* (DETR), which leverages transformers for enhanced feature extraction and detection. Another promising direction involves investigating *Generative Adversary Networks* (GAN) to generate synthetic mitotic figures, addressing the challenge of limited data, particularly for tumor types with few annotated examples. These advancements could enhance the precision and efficiency of mitosis detection, offering valuable support in cancer diagnosis.
